# Surviving stabbing: The physiology of knife crime

**DOI:** 10.1113/EP093241

**Published:** 2025-09-14

**Authors:** Hugh Montgomery, Mike Tipton

**Affiliations:** ^1^ Division of Medicine University College London London UK; ^2^ Human & Applied Physiology, Extreme Environments Laboratory University of Portsmouth Portsmouth UK

## 1 INTRODUCTION

Knife crime is frequent and commonly fatal in both fiction and real life. Perhaps driven in part by the ‘slasher’ and ‘swashbuckler’ movie genres, injury with a blade is in fact the commonest cause of movie death (https://goodluckmate.com/deadliest‐killers). In real life in 2025, nearly 10,000 people died from stab wounds in South Africa; 9000 in Brazil; 8000 in India, 4000–5000 in each of Mexico China, and Russia; and nearly 1800 in the USA (https://worldpopulationreview.com/country‐rankings/stabbing‐deaths‐by‐country). In the year to March 2024, there were 50,500 offenses involving a sharp implement in the UK, and 262 people were murdered by stabbing (https://commonslibrary.parliament.uk/research‐briefings/sn04304/). Low price and ready concealment no doubt contribute to driving such mortality prevalence, as may socioeconomic factors. But it may also be that some assailants merely intended to inflict a superficial wound or to scare. So why *is* it so easy to kill with a blade, and what are the physiological processes involved?

## 2 DIRECT ORGAN INJURY

Occasionally, death may result from the disruption of the function of a vital organ: with sufficient force, for instance, the spinal cord can be severed or the brain badly damaged. Injury to the intestines can cause faecal matter to enter the abdominal cavity, causing subsequent sepsis and circulatory failure (septic shock). However, death more commonly results from injury to the heart or lungs, or from haemorrhage.

## 3 HAEMORRHAGE

The majority of fatal single stab wounds involve a blood vessel or a highly vascular organ (Berg von Linde et al., 2024). In part, this relates to the thin walls of such structures and to their proximity to the skin. Whilst depths vary with body habitus, the free wall of the right ventricle (RV) is only 3–5 mm thick, and lies typically within 1 mm of, or in direct contact with, the chest wall. The overlying parasternal intercostal muscle is typically <3 mm thick on expiration (Gandevia et al., 2006). The wall of the common carotid artery is perhaps 1 mm thick, and its bifurcation lies only 2–4 cm below the skin (Hadley et al., 2005). The popliteal artery wall is <0.8 mm thick and often lies 1–4 cm beneath the skin behind the knee (Olowoyeye et al., 2017). Similarly, the capsule of the (highly vascular) liver is only 2–2.5 cm below the skin, and that of the spleen is 1.5–3 cm deep. Even the abdominal aorta often lies within the range of a standard kitchen knife from the anterior abdominal wall.

Once penetrated, blood will be lost from the vascular structure. Venous pressure varies depending on the state of venous capacitance, tone and circulatory filling but is generally much lower than that in the arterial circulation (e.g., 0–5 mmHg in the inferior vena cava (IVC) and a mean of perhaps 95 mmHg [120/80 mmHg systolic/diastolic] in the adjacent descending aorta). Right ventricular pressures are perhaps 25/4 mmHg, compared to 120/8 mmHg in the left ventricle. However, one should remember that blood *flow* through each cardiac chamber is essentially the same, just as are the flows through the IVC and aorta. These are not insubstantial, with IVC flows at rest ranging from 15 to 37 mL/s (Joseph et al., 2020). Whilst loss from small holes can, in theory, be reduced by natural local activation of the clotting cascade, the efficacy of such clotting is dramatically reduced when deep body temperature is low or falls (as it may in a lightly dressed or exposed individual, or when ‘cold’ blood is transfused); when massive transfusion occurs with a paucity of administration of clotting factors; and when acidosis (which impairs thrombin generation) occurs (common in circulatory shock).

Mortality from injury to a vascular structure thus relates to the structure injured and to the scale of that injury. In part, this is due to the variable ability to directly (or upstream) compress the vascular structure itself. Thus, the femoral artery is readily compressed in the groin, while a tourniquet applied to the upper arm (above arterial pressure) can prevent haemorrhage from a (elbow) brachial artery. However, the subclavian artery (hidden behind the collar bone) or the iliac artery (within the abdominal cavity) cannot be readily compressed.

In part, mortality is also influenced by varying physiological consequences. A stab wound to the right ventricle, for instance, can cause death from haemorrhagic shock, or from blood accumulating in the (poorly distensible) pericardial sac which surrounds it, progressively compressing the RV itself and limiting diastolic filling. More commonly, however, death results from blood loss. Falling arterial pressure decreases activation of arterial baroreceptors in the carotid sinus and aortic arch, thereby stimulating a sympathetic nervous system response. This leads to venoconstriction (thus increasing cardiac filling by reducing vascular capacitance, and – via the Frank–Starling mechanism – increasing cardiac stroke volume), positive cardiac inotropy (greater ejected cardiac volume for any given filling pressure), cardiac chronotropy (increased cardiac cycles per minute) and vasoconstriction (raising arterial pressure for any given cardiac output). Such responses compensate for haemorrhage by maintaining arterial pressure until perhaps one‐third of circulating volume has been lost, at which point partial withdrawal of the sympathetic response can lead to a fall in blood pressure and heart rate (Thomas and Dixon, 2004). Unless haemorrhage is arrested and circulating volume (and oxygen carrying capacity) restored, death may result when perfusion pressure drops below that of autoregulation in core organs, and when the metabolic demands of tissues can no longer be met.

## 4 PULMONARY INJURY

The lung lies immediately beneath the inner rib surface and, like the RV, is thus readily injured by blade penetration. It is a highly vascular structure and death may result from haemorrhage. However, damage to the aerated pulmonary tissue may result in air entering the (usually negatively pressured) pleural space surrounding the lung, followed by accumulation of air outside the lung, and partial or complete collapse of the lung itself. This activates deflation‐activated receptors, driving an increase in vagal activity, which increases respiratory drive (the Herring–Breuer deflation reflex) (Yu, 2018). The collapsed lung is poorly aerated and, whilst alveolar blood flow may fall due to changes in lung parenchymal geometry and pressure (Christophe et al., 2012) as well as to hypoxic pulmonary vasoconstriction (Dunham‐Snary et al., 2017), ventilation–perfusion mismatching (Petersson and Glenny, 2014) still leads to hypoxaemia.

In ‘tension’ (unlike ‘simple’) pneumothorax, the chest wall injury (and/or lung surface injury) acts as a one‐way valve, allowing air to enter the pleural cavity with each breath, but not to escape. This causes a progressive rise in pressure within the pleural cavity and, with it, compression of the affected lung. Initially, the physiological responses are similar to those of a simple pneumothorax. However, rising pleural pressures then lead to compression of the mediastinum, and thus heart and great vessels, shifting them contralaterally across the hemithorax and compromising venous return. Cardiac output thus falls, and death can result from low cardiac output and hypoxia.

## 5 SO, WHAT IS TO BE DONE?

It is thus remarkably easy to kill with a blade, whether accidentally or deliberately. The first requirement is for knives to be removed from our streets, and for those knives available to be less hazardous. Whilst removal of large ‘zombie’ knives from sale has been much vaunted in the UK, it is likely that restricting the sale of pointed blades (as has been advocated: https://www.independent.co.uk/news/uk/home‐news/hugh‐fearnleywhittingstall‐idris‐elba‐keir‐starmer‐good‐morning‐britain‐luther‐b2695958.html) would be far more effective: tissue penetration is hard with a round‐ended blade.

Education is also important. Whilst avoiding giving ‘instruction on how to best kill’, a broader education programme in schools might raise awareness of how easy it is to kill while perhaps only intending to ‘scare’ or ‘defend’. Likewise, a reduction in onscreen violence, or a better representation of its likely consequences, might play a role.

The location of the injury may require rapid and skilled responses in order to save life (for instance, in cardiac or subclavian arterial injury). However, simple first‐aid training (understanding how and where to apply pressure to a bleeding vessel; how to improvise and apply a tourniquet) might also have a great impact. It might also be that tourniquets could be co‐located with automatic external defibrillators, allowing their application by suitably trained individuals or under instruction from emergency service personnel.

## AUTHOR CONTRIBUTIONS

Both authors have read and approved the final version of this manuscript and agree to be accountable for all aspects of the work in ensuring that questions related to the accuracy or integrity of any part of the work are appropriately investigated and resolved. All persons designated as authors qualify for authorship, and all those who qualify for authorship are listed.

### CONFLICT OF INTEREST

The authors have no conflicts of interest.

### FUNDING INFORMATION

This work was not funded.
